# Supplementary material for: A deep learning diagnostic platform for diffuse large B-cell lymphoma with high accuracy across multiple hospitals
Source: Nat Commun. 2020 Nov 26;11:6004. doi: 10.1038/s41467-020-19817-3 (PMC7691991; doi:10.1038/s41467-020-19817-3)
Supplement: Supplementary file 1 — Supplementaty Information [file 41467_2020_19817_MOESM1_ESM.pdf]

Annotations of WSI

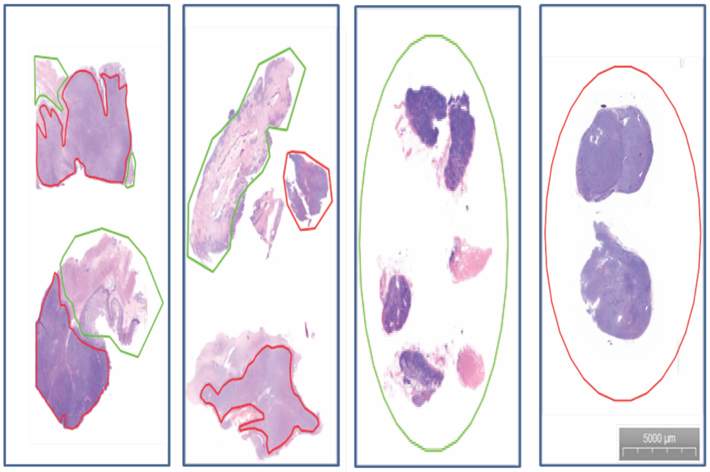

Supplementary Fig. 1. Additional image variations derived from tissue preparation procedures. The scanned whole slide images contained human tissues unrelated to DLBCL. Red lines indicate tissues that contain DLBCL cells, and green lines indicate tissues unrelated to DLBCL.

Supplementary Table 1. Comparison of analytical results for the CIFAR-10 data

Result (% accuracy)	Method	Venue
96.88%	SDL global optimization (in this study)	SDL 2020
96.53%	Fractional Max-Pooling	arXiv 2015
95.59%	Striving for Simplicity: The All Convolutional Net	ICLR 2015
94.16%	All you need is a good init	ICLR 2016
94%	Lessons learned from manually classifying CIFAR-10	unpublished 2011
93.95%	Generalizing Pooling Functions in Convolutional Neural Networks: Mixed, Gated, and Tree	AISTATS 2016
93.72%	Spatially-sparse convolutional neural networks	arXiv 2014
93.63%	Scalable Bayesian Optimization Using Deep Neural Networks	ICML 2015
93.57%	Deep Residual Learning for Image Recognition	arXiv 2015
93.45%	Fast and Accurate Deep Network Learning by Exponential Linear Units	arXiv 2015
93.34%	Universum Prescription: Regularization using Unlabeled Data	arXiv 2015
93.25%	Batch-normalized Maxout Network in Network	arXiv 2015
93.13%	Competitive Multi-scale Convolution	arXiv 2015
92.91%	Recurrent Convolutional Neural Network for Object Recognition	CVPR 2015

Result (% accuracy)	Method	Venue
92.49%	Learning Activation Functions to Improve Deep Neural Networks	ICLR 2015
92.45%	cifar.torch	unpublished 2015
92.40%	Training Very Deep Networks	NIPS 2015
92.23%	Stacked What-Where Auto-encoders	arXiv 2015
91.88%	Multi-Loss Regularized Deep Neural Network	CSVT 2015
91.78%	Deeply-Supervised Nets	arXiv 2014
91.73%	BinaryConnect: Training Deep Neural Networks with binary weights during propagations	NIPS 2015
91.48%	On the Importance of Normalisation Layers in Deep Learning with Piecewise Linear Activation Units	arXiv 2015
91.40%	Spectral Representations for Convolutional Neural Networks	NIPS 2015
91.2%	Network In Network	ICLR 2014
91.19%	Speeding up Automatic Hyperparameter Optimization of Deep Neural Networks by Extrapolation of Learning Curves	IJCAI 2015
90.78%	Deep Networks with Internal Selective Attention through Feedback Connections	NIPS 2014
90.68%	Regularization of Neural Networks using DropConnect	ICML 2013

Result (% accuracy)	Method	Venue
90.65%	Maxout Networks	ICML 2013
90.61%	Improving Deep Neural Networks with Probabilistic Maxout Units	ICLR 2014
90.5%	Practical Bayesian Optimization of Machine Learning Algorithms	NIPS 2012
89.67%	APAC: Augmented PAttern Classification with Neural Networks	arXiv 2015
89.14%	Deep Convolutional Neural Networks as Generic Feature Extractors	IJCNN 2015
89%	ImageNet Classification with Deep Convolutional Neural Networks	NIPS 2012
88.80%	Empirical Evaluation of Rectified Activations in Convolution Network	ICML workshop 2015
88.79%	Multi-Column Deep Neural Networks for Image Classification	CVPR 2012
87.65%	ReNet: A Recurrent Neural Network Based Alternative to Convolutional Networks	arXiv 2015
86.70 %	An Analysis of Unsupervised Pre-training in Light of Recent Advances	ICLR 2015
84.87%	Stochastic Pooling for Regularization of Deep Convolutional Neural Networks	arXiv 2013

Result (% accuracy)	Method	Venue
84.4%	Improving neural networks by preventing co-adaptation of feature detectors	arXiv 2012
83.96%	Discriminative Learning of Sum-Product Networks	NIPS 2012
82.9%	Stable and Efficient Representation Learning with Nonnegativity Constraints	ICML 2014
82.2%	Learning Invariant Representations with Local Transformations	ICML 2012
82.18%	Convolutional Kernel Networks	arXiv 2014
82%	Discriminative Unsupervised Feature Learning with Convolutional Neural Networks	NIPS 2014
80.02%	Learning Smooth Pooling Regions for Visual Recognition	BMVC 2013
80%	Object Recognition with Hierarchical Kernel Descriptors	CVPR 2011
79.7%	Learning with Recursive Perceptual Representations	NIPS 2012
79.6 %	An Analysis of Single-Layer Networks in Unsupervised Feature Learning	AISTATS 2011
78.67%	PCANet: A Simple Deep Learning Baseline for Image Classification?	arXiv 2014
75.86%	Enhanced Image Classification With a Fast-Learning Shallow Convolutional Neural Network	arXiv 2015
