# Supplementary material for: A deep learning diagnostic platform for diffuse large B-cell lymphoma with high accuracy across multiple hospitals
Source: Nat Commun. 2020 Nov 26;11:6004. doi: 10.1038/s41467-020-19817-3 (PMC7691991; doi:10.1038/s41467-020-19817-3)
Supplement: Supplementary file 2 — Reporting Summary [file 41467_2020_19817_MOESM2_ESM.pdf]

nature research

Corresponding author(s): Shaoguang Li

Last updated by author(s): Oct 19, 2020

Reporting Summary

Nature Research wishes to improve the reproducibility of the work that we publish. This form provides structure for consistency and transparency in reporting. For further information on Nature Research policies, see our Editorial Policies and the Editorial Policy Checklist.

Statistics

For all statistical analyses, confirm that the following items are present in the figure legend, table legend, main text, or Methods section.

n/a	Confirmed	
[ ]	[x]	The exact sample size (*n*) for each experimental group/condition, given as a discrete number and unit of measurement
[x]	[ ]	A statement on whether measurements were taken from distinct samples or whether the same sample was measured repeatedly
[x]	[ ]	The statistical test(s) used AND whether they are one- or two-sided<br>*Only common tests should be described solely by name; describe more complex techniques in the Methods section.*
[x]	[ ]	A description of all covariates tested
[x]	[ ]	A description of any assumptions or corrections, such as tests of normality and adjustment for multiple comparisons
[x]	[ ]	A full description of the statistical parameters including central tendency (e.g. means) or other basic estimates (e.g. regression coefficient) AND variation (e.g. standard deviation) or associated estimates of uncertainty (e.g. confidence intervals)
[x]	[ ]	For null hypothesis testing, the test statistic (e.g. *F*, *t*, *r*) with confidence intervals, effect sizes, degrees of freedom and *P* value noted<br>*Give P values as exact values whenever suitable.*
[x]	[ ]	For Bayesian analysis, information on the choice of priors and Markov chain Monte Carlo settings
[x]	[ ]	For hierarchical and complex designs, identification of the appropriate level for tests and full reporting of outcomes
[x]	[ ]	Estimates of effect sizes (e.g. Cohen's *d*, Pearson's *r*), indicating how they were calculated

Our web collection on statistics for biologists contains articles on many of the points above.

Software and code

Policy information about availability of computer code

Data collection	No software was used.
Data analysis	In this study, we used the following commercial software: Matlab2019b with Deep Learning Toolbox and Image Processing Toolbox.

For manuscripts utilizing custom algorithms or software that are central to the research but not yet described in published literature, software must be made available to editors and reviewers. We strongly encourage code deposition in a community repository (e.g. GitHub). See the Nature Research guidelines for submitting code & software for further information.

Data

Policy information about availability of data

All manuscripts must include a data availability statement. This statement should provide the following information, where applicable:

- Accession codes, unique identifiers, or web links for publicly available datasets
- A list of figures that have associated raw data
- A description of any restrictions on data availability

Data availability. We present three illustrative medical image analysis applications built using GOTDP-MP-CNNs infrastructure. All of the raw image data can be downloaded from www.umass.edu/AI/data upon request to shaoguang.li@umassmed.edu.
This paper was produced using no publicly available DLBCL imaging data except the experimental CIFAR-10 data. The authors have made every effort to make available links to these resources as well as make publicly available the software methods and information used to produce the datasets, analyses, and summary information. Further information on research design is available in the Nature Research Reporting Summary linked to this article. All data supporting the findings of this study are available within the paper or from https://fts.umassmed.edu (user name: dli; password: Dong1956) or from the corresponding author upon reasonable request to shaoguang.li@umassmed.edu. The size of our research data is too huge to be properly accepted and stored in public repositories. Also, due to the complexity of our research data, it is better for public users to reach out to shaoguang.li@umassmed.edu for avoiding any misunderstanding of the data and

for using the data appropriately.

Code availability. Some of source code can be found from https://fts.umassmed.edu (user name: dli; password: Dong1956) or obtained by sending a request to shaoguang.li@umassmed.edu.

Field-specific reporting

Please select the one below that is the best fit for your research. If you are not sure, read the appropriate sections before making your selection.

- [x] Life sciences
- [ ] Behavioural & social sciences
- [ ] Ecological, evolutionary & environmental sciences

For a reference copy of the document with all sections, see nature.com/documents/nr-reporting-summary-flat.pdf

Life sciences study design

All studies must disclose on these points even when the disclosure is negative.

Sample size	A unique feature of our study is to use sample size that is much less than the sample size published by others in the AI-related studies. Our goal was to use as small as possible the sample size but still maintain high diagnostic accuracy for DLBCL. Thus, the sample size in our study was determined based on our analysis of tissue images, aiming to go as small as possible using published information as a reference. From the papers we cited in our manuscript, commonly-used sample size published in AI-related studies is shown.
Data exclusions	Data exclusions were not done in our study.
Replication	In our study, we used our deep learning platform with multiple pre-trained CNNs (GOTDP-MP-CNNs) to read and diagnose DLBCL tissue images in three separate hospitals and achieved high diagnostic accuracy in all these hospitals. Thus, the experiments were performed independently three times with similar results, showing the reproducibility of our experiments.
Randomization	The samples were allocated into experimental groups randomly.
Blinding	The investigators involved in this study were blinded to group allocation during data collection and data analysis.

Reporting for specific materials, systems and methods

We require information from authors about some types of materials, experimental systems and methods used in many studies. Here, indicate whether each material, system or method listed is relevant to your study. If you are not sure if a list item applies to your research, read the appropriate section before selecting a response.

Materials & experimental systems

n/a	Involved in the study	
x		Antibodies
x		Eukaryotic cell lines
x		Palaeontology and archaeology
x		Animals and other organisms
x		Human research participants
x		Clinical data
x		Dual use research of concern

Methods

n/a	Involved in the study	
x		ChIP-seq
x		Flow cytometry
x		MRI-based neuroimaging
